# The Effectiveness of Lifestyle Triple P in the Netherlands: A Randomized Controlled Trial

**DOI:** 10.1371/journal.pone.0122240

**Published:** 2015-04-07

**Authors:** Sanne M. P. L. Gerards, Pieter C. Dagnelie, Jessica S. Gubbels, Stef van Buuren, Femke J. M. Hamers, Maria W. J. Jansen, Odilia H. M. van der Goot, Nanne K. de Vries, Matthew R. Sanders, Stef P. J. Kremers

**Affiliations:** 1 Department of Health Promotion, and NUTRIM School of Nutrition and Translational Research in Metabolism, Maastricht University, Maastricht, the Netherlands; 2 Department of Epidemiology, Maastricht University, Maastricht, the Netherlands; 3 CAPHRI School of Public Health and Primary Care, Maastricht University, Maastricht, the Netherlands; 4 CARIM School for Cardiovascular Diseases, Maastricht University, Maastricht, the Netherlands; 5 Netherlands Organization for Applied Scientific Research (TNO), Leiden, the Netherlands; 6 Department of Methodology and Statistics, University of Utrecht, Utrecht, the Netherlands; 7 Academic Collaborative Center for Public Health Limburg, Public Health Services, Geleen, the Netherlands; 8 Department of Youth Health Care, Public Health Services, Geleen, the Netherlands; 9 Parenting and Family Support Centre, School of Psychology, The University of Queensland, Brisbane, Australia; Weill Cornell Medical College Qatar, QATAR

## Abstract

**Introduction:**

Lifestyle Triple P is a general parenting intervention which focuses on preventing further excessive weight gain in overweight and obese children. The objective of the current study was to assess the effectiveness of the Lifestyle Triple P intervention in the Netherlands.

**Method:**

We used a parallel randomized controlled design to test the effectiveness of the intervention. In total, 86 child-parent triads (children 4–8 years old, overweight or obese) were recruited and randomly assigned (allocation ratio 1:1) to the Lifestyle Triple P intervention or the control condition. Parents in the intervention condition received a 14-week intervention consisting of ten 90-minute group sessions and four individual telephone sessions. Primary outcome measure was the children’s body composition (BMI z-scores, waist circumference and skinfolds). The research assistant who performed the measurements was blinded for group assignment. Secondary outcome measures were the children’s dietary behavior and physical activity level, parenting practices, parental feeding style, parenting style, and parental self-efficacy. Outcome measures were assessed at baseline and 4 months (short-term) and 12 months (long-term) after baseline. Multilevel multiple regression analyses were conducted to determine the effect of the intervention on primary and secondary outcome measures.

**Results:**

No intervention effects were found on children’s body composition. Analyses of secondary outcomes showed positive short-term intervention effects on children’s soft-drink consumption and parental responsibility regarding physical activity, encouragement to eat, psychological control, and efficacy and satisfaction with parenting. Longer-term intervention effects were found on parent’s report of children’s time spent on sedentary behavior and playing outside, parental monitoring food intake, and responsibility regarding nutrition.

**Conclusion:**

Although the Lifestyle Triple P intervention showed positive effects on some parent reported child behaviors and parenting measures, no effects were visible on children’s body composition or objectively measured physical activity. Several adjustments of the intervention content are recommended, for example including a booster session.

**Trial Registration:**

Nederlands Trial Register NTR 2555

## Introduction

In response to the increasing prevalence of overweight and obesity among children worldwide [1], childhood obesity intervention programs are being developed and evaluated on a large scale [2]. The importance of involving parents in such interventions is increasingly recognized [2–4]. Intervention studies involving parents predominantly aim to change *parenting practices*, specific parenting behaviors relating to children’s eating and/or physical activity, for example increasing fruit availability at home and encouraging a child to be physically active. Intervention studies focusing on parenting practices have shown promising results, although effects in the longer term have been limited [3].

Another relevant aspect of parenting that influences children’s weight-related health outcomes is *general parenting* or *parenting styles* [5]. General parenting is defined as the emotional climate in which parenting takes place, encompassing parent-child interactions across situations [6]. Different dimensions of parenting can be distinguished, such as the extent to which parents respond to their children’s needs (responsiveness) and the extent to which parents exert control over their children’s behaviors (demandingness). Authoritative parenting (both responsive and demanding) seems to have beneficial effects on children’s nutritional intake, physical activity levels, and BMI [5]. Next to responsiveness and demandingness, a third relevant dimension of parenting is psychological control [7], the extent to which parents regulate their children’s behavior through psychological means, for example by guilt induction and love withdrawal.

An added value of targeting at the broader concept of parenting style rather than only parenting practices, is that it may lead to more sustained behavior change and a broader public health effect [8] (i.e. by impacting on a broad range of child outcomes such as reduced substance abuse and academic performance [9]). Additionally, parenting practices may be more effective when embedded in a positive parenting environment [10,11]. Targeting parenting styles seems effective in preventing or treating childhood obesity [8,12–14], and improving child and parenting outcomes such as children’s eating behaviors [14], children’s physical activity levels [13], parental feeding styles [15], parenting practices [15], and general parenting [12].

Lifestyle Triple P is a derivative of the Triple P Positive Parenting Program [16], a multi-level parenting and family support strategy. Lifestyle Triple P is tailored to the concerns of parents of overweight and obese children and aims at changing both parenting practices and general parenting styles. Its efficacy has been tested in a randomized controlled trial (RCT) in Australia [17]. Children of parents who participated in the intervention had a reduced BMI z-score relative to the wait-list control group [17]. Furthermore, the intervention increased parental confidence in managing children’s weight-related problem behaviors and improved parenting styles.

In view of the lack of evidence-based childhood obesity prevention interventions in the Netherlands, we conducted an RCT evaluating the effectiveness of Lifestyle Triple P in the Netherlands [18]. The aim of the current study was to assess the effectiveness of the Lifestyle Triple P intervention, primarily in terms of prevention of excessive weight gain in overweight and obese children. The primary outcome was the children’s body composition (measured by BMI z-score, waist circumference and skinfolds). Secondary outcome measures were the children’s dietary behavior and physical activity level, parenting practices, parental feeding style, parenting style, and parental self-efficacy.

## Materials and Methods

### Design

A parallel-group randomized controlled trial (allocation ratio 1:1) was conducted in the southern part of the province of Limburg, the Netherlands in 2010–2013.

The Medical Ethics Committee of the University Hospital Maastricht and Maastricht University approved the study protocol (reference number NL 31988.068.10 / MEC 10-3-052). This trial is registered in the Netherlands Trial Registry (NTR 2555). The protocol of this trial and supporting CONSORT checklist are available as supporting information: see S1 Checklist and S1 Protocol.

### Participants

Eligible participants were parent-child triads. Parents of children aged between 4 and 8 years were eligible for participation if their child was considered to be overweight or obese at inclusion, based on the BMI, using the international sex- and age-specific cut-off points proposed by Cole et al [19]. Furthermore, eligible parents were living in the southern part of Limburg, and were able to communicate in Dutch. Parents who agreed to participate and who both signed the informed consent form were included in the study.

Four different recruitment strategies were used to recruit participants. First, professionals working in the Dutch youth health care system (YHC: a preventive health care system available for all children aged 0–19 years) were asked to refer parents of overweight or obese children to the Lifestyle Triple P Intervention. Second, parents whose children were overweight according to the YHC medical records and other research projects were actively approached for participation in the intervention. Third, a mass media campaign (brochure, poster, advertisements in newspapers and website) was used to inform parents about the intervention and ask them to register for it. Finally, invitation letters were sent to parents of primary school children.

Based on the sample size calculation, we needed 84 families (adjusted for attrition and nesting effects) to detect a difference of 0.17 BMIz points (equivalent to approximately 0.30 BMI points among 4-year-olds and 0.50 BMI points among 8-year-olds) between the intervention and control conditions [20] (power of 0.9 and P<0.05). In total, we enrolled 86 parent-child triads (44 intervention triad, 42 control triads) from December 2010 until February 2012.

### Randomization

After baseline measures, parents were randomly allocated to the intervention or control condition. The randomization scheme was generated by an independent researcher (PCD) who was not directly involved in data collection or intervention delivery, using a block size of four (allocation ratio 1:1) and sealed envelopes. The randomization was concealed to all other members of the study team. A member of the study team (SMPLG) phoned the research institute in the presence of the parents to receive the group allocation.

### Intervention delivery

Parents who were assigned to the intervention condition received the Lifestyle Triple P intervention, a 14-week intervention comprising ten 90-minute parental group sessions and four individual 15–30 minute telephone sessions. Both parents were invited to attend the group sessions. The intervention was delivered to parents-only. The group sessions took place at three different locations of the Public Health Services in South Limburg (Maastricht, Heerlen and Geleen). Per location, two intervention groups of parents were formed, the group size ranged from 5 to 10 parents.

Lifestyle Triple P is an intervention strategy consisting of active skills training methods based on self-regulation principles. Parents were instructed on a range of nutrition, physical activity and positive parenting strategies. Individual telephone sessions provided parents individual support in implementing the strategies at home. The intervention was led by three different Lifestyle Triple P facilitators. These health professionals have been accredited after attending an official 3-day Triple P training course and an additional Lifestyle Triple P day. The intervention materials consisted of a parent workbook, a recipe book, and an active games booklet, all translated from English into Dutch for the current study, by Triple P International. The Lifestyle Triple P intervention was developed by the University of Queensland in Brisbane, Australia [21]. For a more detailed description of the intervention we refer to an earlier publication [18].

### Control condition

Parents who were assigned to the control condition received two brochures (one on healthy nutrition and physical activity and one on positive parenting), as well as a short knowledge quiz via the Internet (sent via email) including tailored advice and suggestions for active exercises at home.

### Measures

Outcome measures were assessed at baseline (March 2011 till March 2012), at 4 months (immediately after the intervention; June 2011 till Sept 2012), and at 12 months (March 2012 till February 2013).

Anthropometric baseline measurements were started as soon as enough participants (a minimum of 10 parent-child triads) per site (Heerlen, Geleen or Maastricht) had been recruited. At each site, two waves of baseline measurements took place. All the anthropometric measurements took place during a visit to the Public Health Service offices by a YHC professional who was blinded for group allocation, using a standardized protocol. At the end of these visits, parents received a Dutch questionnaire and children received an Actigraph accelerometer. They were instructed to send these materials back by mail.

### Primary outcome measures

The primary outcome measure was the children’s body composition, operationalized as BMI z-score, waist circumferences, and biceps and triceps skinfolds. Weight was measured using an electronic portable scale (standardized Seca 899) to the nearest 0.1 kg while the child was only wearing underwear. Height was measured using a portable stadiometer (Seca 214) with an accuracy of 1 mm. Weight and height was used to calculate Body Mass Index (BMI). BMI was then recoded into BMI z-scores standardized for age and gender, based on a national reference population (i.e. the Fourth Dutch National Growth Study) [22]. We recoded weight status into three different categories, based on BMI z-scores [23]: normal weight (5^th^–84^th^ percentile, BMI z-scores: -1.65 to 1.04), overweight (85^th^–95^th^ percentile, BMI z-scores: 1.05 to 1.64), obese (≥95^th^ percentile BMI z-scores: ≥1.65). Waist circumference was measured with a flexible tape to the nearest 1 mm. In addition, biceps and triceps skinfold thickness was measured to the nearest 0.1 mm using a Harpenden skinfold caliper. Each skinfold was measured three times. If the scores differed by more than 10%, three extra skin-folds were measured. The median of the three or six measurements was calculated. The sum score of the biceps and triceps skinfolds was calculated and used for data analyses.

### Secondary outcome measures

#### Children’s diet and physical activity level

Children’s dietary intake was assessed using several items from a validated Food Frequency Questionnaire (FFQ) designed to accurately assess energy intake of Dutch children aged 2–12 years [24,25]. The validation study showed a correlation coefficient between the original questionnaire and the doubly labeled water method of 0.62. We measured the frequency of their child’s having breakfast and having snacks, and the frequency and amount of their child’s consumption of fruits, vegetables, soft drinks (including sugar-sweetened beverages), and water. The frequency was measured in days per week. The amount was measured in pieces (fruit), grams (vegetables), and glasses (soft-drink and water). The number of days and the amount were then multiplied to calculate the amount per week.

Children’s physical activity level was assessed using an objective (accelerometer) and a subjective outcome measure (questionnaire for parents). Children were asked to wear an Actigraph accelerometer (GT1M, Actigraph, Pensacola, Florida) for 7 consecutive days, in the week following the anthropometric measurements. Children were instructed to only remove the accelerometer at night, while they were taking a bath or shower and while they were swimming. Measurements that included at least 2 weekdays and 1 weekend day (daily wear time ≥600 minutes) were considered valid and were used in the analyses. Periods of ≥90 minutes of non-wearing time (defined as consecutive zero counts) were removed from the data [27]. The time interval or epoch was set at 15 sec. The thresholds proposed by Evenson et al. [28] were used to distinguish different intensities of physical activity: sedentary behavior (≤25 counts per epoch), light physical activity (26–573 counts per epoch), moderate physical activity (574–1002 counts per epoch), and vigorous physical activity (≥1003 counts per epoch). We combined moderate and vigorous physical activity into one category.

In addition, parents were asked to indicate the frequency (days per week) and duration (number of hours) of several physical activity behaviors [26]: TV watching, computer games (combined into one scale, i.e., sedentary behavior), playing outside, sports club attendance, and use of active transport (walking/cycling to and from school). The number of days and duration were multiplied to calculate the number of hours spent on a particular activity per week.

#### Parenting behaviors

Parenting practices were measured using two scales of the Child Feeding Questionnaire [29] (see Table 1 for detailed information about the scales). The *responsibility regarding nutrition* scale consisted of two items assessing parents’ responsibility regarding their child’s feeding. The *monitoring food intake* scale assessed the extent to which parents oversaw their child's eating behavior.

**Table 1:** Overview of parenting scales.

Scale	N items	Cronbach’s Alpha	Example of items
*Parenting practices* ^*a*^			
Monitoring food intake	4	0.76	How often do you keep track of the sweets and the snack food that your child eats?
Responsibility regarding nutrition	2	0.58	How often are you responsible for deciding your child’s portion sizes?
Monitoring physical activity	2	0.67	How often do you keep track of the amount of physical activity your child engages in?
Responsibility regarding physical activity	2	0.91	How often are you responsible for deciding whether your child gets enough physical activity?
*Feeding styles* ^*b*^			
Control over eating	10	0.73	I allow my child to choose which foods to have for meals.
Instrumental feeding	4	0.69	In order to get my child to behave him/herself I promise him/her something to eat.
Emotional feeding	5	0.83	I give my child something to eat to make him/her feel better when he/she is feeling upset.
Encouragement to eat	8	0.76	I encourage my child to taste foods that he/she has not tasted before
*General parenting*			
Authoritative parenting^c^	12	0.61	I respect my child’s opinion and encourage him/her to express it.
Authoritarian parenting^c^	12	0.62	I believe children should not have secrets from their parents.
Psychological control^c^	8	0.60	I avoid looking at my child if he/she does not see things my way.
Efficacy of and satisfaction with parenting^d^	16	0.76	Sometimes I feel I’m not getting anything done.

^a^5-point Likert scale form disagree to agree

^b^5-point Likert scale from never to always

^c^5-point Likert sc^a^le ^from^ completely disagree to completely agree

^d^6-pont Likert scale from strongly disagree to strongly agree.

In addition, we used CFQ items which were converted to the physical activity context, the ‘Physical Activity-Related Parenting Questionnaire’ [26]: *responsibility regarding physical activity* (parents’ perception of their responsibility for their child’s physical activity level), and *monitoring physical activity* (the extent to which parents check their child’s physical activity level).

The validated Dutch version [30] of the Parental Feeding Style Questionnaire [31] was used to measure four different feeding styles: *instrumental feeding* (using food as a reward), *emotional feeding* (feeding in response to emotional distress), *encouragement to eat* (encouraging food variety and interest in food), and *control over eating* (parental restrictions; see Table 1).

A validated Dutch version [32] of the Child Rearing Practices Report [33] was included to assess parents’ child-rearing attitudes, values, behaviors, and goals. The questionnaire can be used to distinguish two different parenting styles: *authoritative parenting* and *authoritarian parenting* (see Table 1). Items on the authoritarian scale reflect ‘the frequent use of physical punishment, verbal reprimands, prohibitions, discouragement of the child’s expression, emphasis on fear of external consequences of transgression and strict supervision of child’ [32]. The authoritative scale consists of items ‘indicating the emphasis on inductive methods, reasoning with the child, appreciation of the child’s accomplishments, fostering the child’s individuality, and encouraging open communication between parents and the child regarding both positive and negative feelings’[32]. Additionally, we included items from a Dutch version of the validated *psychological control* scale [34].

The validated ‘Being a Parent Scale’ [35] was used to assess parenting self-esteem, which consists of parental self-efficacy and their satisfaction derived from parenting. All individual items were combined into one scale measuring parenting self-esteem (total score on the Being a Parent Scale) [35].

#### Demographics

A range of demographics was included in the baseline questionnaire. Child characteristics assessed included gender and date of birth. Parental characteristics included country of birth of mother and father (recoded into Netherlands vs. other), employment of mother and father (hours of paid work per week), educational level of mother and father and marital status of the parents. Educational level was recoded into low (primary school, lower vocational education, and lower general secondary education), medium (intermediate vocational education, higher general secondary education, and university preparatory education), and high (higher vocational education and university) [36]. Finally, the marital status of the parents was recoded into married or living together vs. other.

Weight and height (in order to calculate BMI) of parents and siblings were also measured, using the same standardized procedures as for the children. If the parents were not willing to undress and were fully clothed, 1 kg was subtracted from the body weight [37]. These measures were conducted at baseline, 4 and 12 months. However, due to lower participants’ rates among siblings and low posttest rates among parents, only the baseline data of the parents were used to correct for in our effect analyses.

#### Process evaluation

We measured parental attendance in the group sessions and participation in the individual telephone sessions. In addition, process evaluation questions were included in the 4-month questionnaire. The Client Satisfaction Questionnaire was administered to measure parent satisfaction with the Triple P intervention [38]. The CSQ consists of 13 items (e.g., ‘To what extent has the program met your needs?’), on a scale from 1–7. Sum scores ranged from 13 to 91, with greater scores indicating greater satisfaction with the intervention (Cronbach’s Alpha = 0.88).

Furthermore, parents were asked to respond to 3 general items on a 5-point scale (‘What is your general impression of the program?’, ‘Do you think the program was interesting’? and ‘Do you think the program was clear?’) and to provide an overall rating for the intervention on a scale from 0–10.

### Statistical analysis

SPSS 19.0 was used for the analyses. In all analyses, p-values <0.05 were considered statistically significant. Descriptive statistics were calculated to describe demographics. The internal consistency of the scales was determined by calculating Cronbach’s alpha. We calculated effect sizes (ES; Cohen’s d) by dividing the change in the outcome measures by the pooled standard deviation of the baseline scores of the study group [39]. Effect sizes were interpreted using the classification defined by Lipsey [40]: small effect (ES≤0.32), medium effect (ES 0.33–0.55), or large effect (ES≥0.56).

Multilevel multiple regression analyses were conducted to determine the effect of the intervention on changes in primary and secondary outcome measures, at 4 and 12 months (short-term and long-term), adjusting for the multilevel structure introduced by the delivery of the intervention in groups. Random coefficients (a random intercept on the group level and a random slope for condition (intervention vs. control) on the group level) were entered using a forward procedure, and were retained in the final model if the -2 log likelihood of the model changed significantly compared to the previous model (Likelihood ratio test).

Subsequently, models were corrected for relevant confounders (regression coefficient of condition changes >10% when the confounder is included in the model), i.e. child’s age, gender, mother’s country of birth, mother’s educational level (low, medium, high), mother’s employment status (hours per week), mother’s BMI and marital status (married or partners living together vs. not). Missing values on covariates were imputed by the group mean (linear variables) or the median (categorical variables).

We performed complete-case analyses using all available data, according to intention-to-treat principles. In addition, we used multiple imputation to assess the impact of missing responses [41], using m = 10. Whereas the complete case analyses are the main focus of the paper, results of the regression analyses in which the multiple imputation approach was used to treat missing values are depicted in S1, S2 and S3 Tables.

## Results

### Response

**Fig 1:**
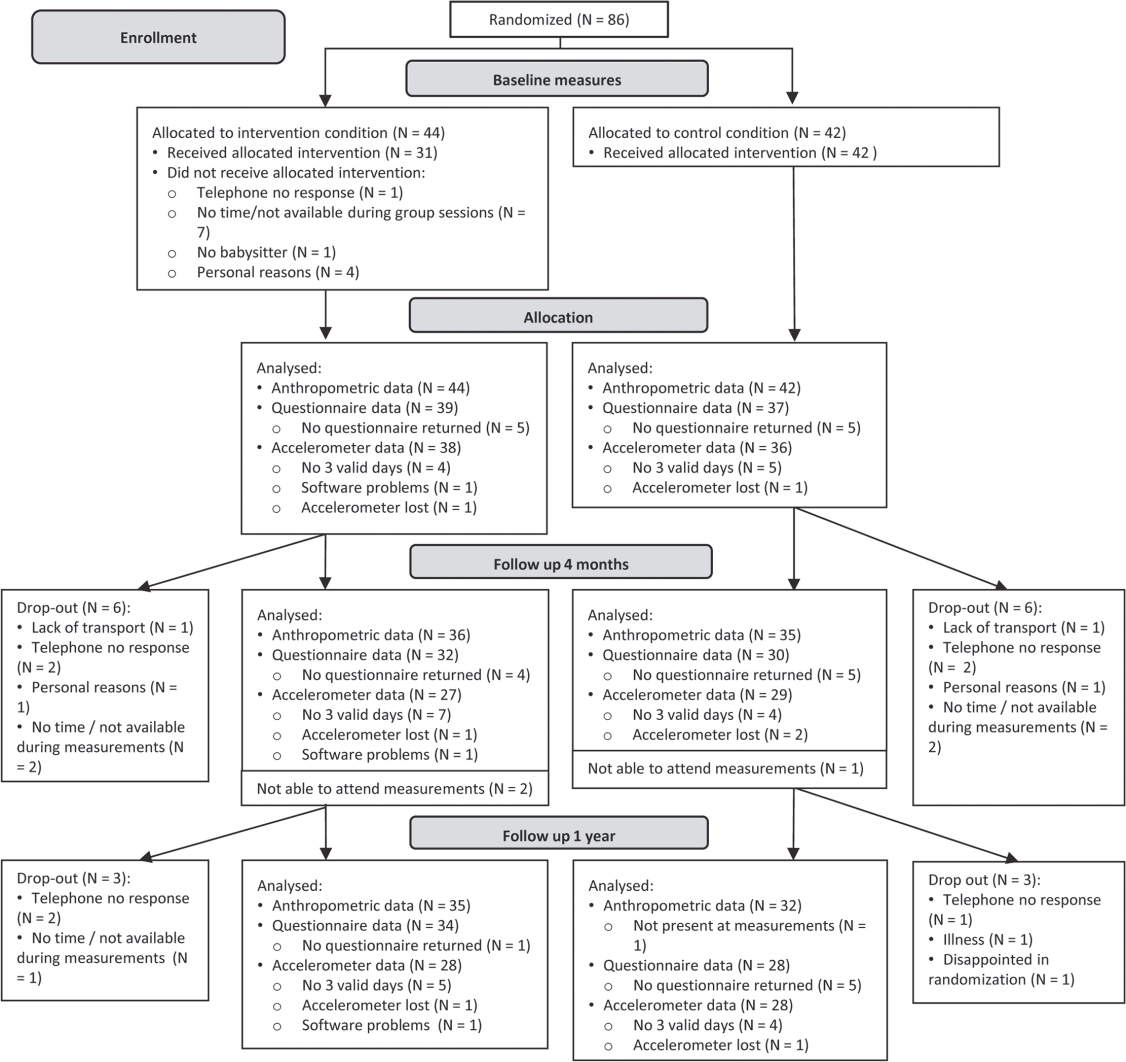
Flow chart of participants. Follow-up and drop-out of participants in the intervention and control conditions.

Of the 86 children who were randomized and underwent baseline anthropometric measurements, 76 (88%) of their parents filled out the baseline questionnaire for the child, and 74 children (86%) had valid baseline accelerometer data, see Fig 1. At 4 months after baseline, 71 children (83%) attended the anthropometric measurements. Parents of 62 children (72%) filled out the 4-months questionnaire, and 56 children (65%) had valid accelerometer data. Twelve months after baseline, 69 children (80%) attended the anthropometric measurements. The final questionnaire was filled out by parents of 62 children (72%) and 56 children (65%) had valid accelerometer data. Drop-out with regard to demographics was non-selective. Baseline characteristics of the children and the parents are depicted in Table 2. Children were on average 7.2 (±1.4) years old, more than half (56%) of the children were female and 63% were obese. Mothers in the intervention condition had a higher mean BMI than those in the control condition (30.19 (±6.71) vs. 26.29(±4.38)).

**Table 2:** Baseline characteristics.

	Variable		Intervention (N = 44)		Control (N = 42)	
			N (%)	Mean±SD	N (%)	Mean±SD
Child	Gender	Male	19 (43.2)		19 (45.2)	
		Female	25 (56.8)		23 (54.8)	
	Age			7.14±1.55		7.29±1.31
	BMI z-score			1.82±0.83		1.87±0.74
	BMI score			20.37±2.79		20.67±3.12
	Weight status	Normal	8 (18.2)		5 (11.9)	
		Overweight	8 (18.2)		11 (26.2)	
		Obesity	28 (63.6)		26 (61.9)	
Parents^1^	Marital status	Married or living together	33 (84.6)		33 (89.2)	
		Other	6 (15.4)		4 (10.8)	
Mother^1^ ^,^ ^2^	Country of birth	Netherlands	33 (84.6)		26 (70.3)	
		Other	6 (15.4)		11 (29.7)	
	Educational level	Low	7 (17.9)		10 (27.0)	
		Medium	20 (51.3)		12 (32.4)	
		High	12 (30.8)		15 (40.5)	
	Employment	(*hours per week*)		17.55±12.93		15.94±15.97
	BMI			30.19±6.71		26.92±4.38
	Weight status	Normal	10 (26.3)		17 (44.7)	
		Overweight	8 (22.2)		13 (34.2)	
		Obesity	18 (47.4)		8 (21.1)	
Fathers^1^ ^,^ ^3^	Country of birth	Netherlands	30 (76.9)		25 (67.5)	
		Other	7 (17.9)		10 (27.0)	
	Educational level	Low	7 (17.9)		8 (21.6)	
		Medium	14 (35.9)		14 (37.8)	
		High	13 (33.3)		12 (32.4)	
	Employment	*(hours per week)*		31.99±15.08		25.11±17.85
	BMI			30.65±5.17		28.68±5.16
	Weight status	Normal	5 (19.2)		6 (26.1)	
		Overweight	7 (26.9)		10 (43.5)	
		Obesity	14 (53.8)		7 (30.4)	

^1^ 39 parents in the intervention condition and 37 parents in the control condition filled out the baseline questionnaire.

^2^ 38 mothers in the intervention condition and 38 mothers in the control condition attended the anthropometric measurement sessions.

^3^ 26 fathers in the intervention condition and 23 fathers in the control condition attended the anthropometric measurement sessions.

## Outcomes

### Primary outcome measure

We did not find any significant intervention effects on children’s BMI z-score, waist circumference, or skinfold thickness at 4 and 12 months after baseline (see Table 3).

**Table 3:** Short- and long term intervention effects on anthropometric outcomes.

	T0 (N = 86^1^)		Change T0-T1 (N = 71^1^)				Change T0-T2 (N = 67^1^)			
	Intervention	Control	Intervention	Control			Intervention	Control		
Variable	Mean±SD	Mean±SD	Mean±SD	Mean±SD	B (95% CI)	Cohen’s d	Mean±SD	Mean±SD	B (95% CI)	Cohen’s d
BMI z-score	1.82±0.83	1.87±0.74	-0.06±0.19	-0.06±0.18	-0.01 (-0.11, 0.08)	0	0.05±0.26	-0.08±0.27	0.11 (-0.03,0.25)	0.24
Waist circumference^2^ ^,^ ^3^	67.23±8.44	68.76±8.68	0.66±2.32	1.04±2.07	-0.79 (-1.85, 0.28)	-0.05	3.88±2.99	3.44±3.46	-0.70 (-2.28, 0.88)	0.07
Skinfolds^2^ ^,^ ^4^	44.21±13.89	43.79±10.96	-1.79±5.00	-2.82±6.32	-0.12 (-2.87, 2.63)	0.18	-0.33±7.94	-0.78±7.68	-1.70 (-5.39, 1.99)	0.06

Note: T0 = baseline, T1 = 4 months after baseline, T2 = 12 months after baseline, SD = standard deviation, B = regression coefficient, CI = confidence interval.

^1^Maximum number of respondents, numbers differ per outcome measure.

^2^Analyses corrected for child’s age and gender.

^3^Waist circumference was measured in cm.

^4^Skinfolds is the sum score of the biceps and triceps skinfolds, measured in mm.

*P<0.05

**P<0.001.

### Secondary outcome measures

#### Children’s nutrition and physical activity level

Regarding children’s physical activity and dietary behavior, all differences between the intervention and control condition with at least a medium effect size were in favor of the intervention condition (Table 4). No significant effects were found for children’s objectively measured physical activity. At 4 months, a significant intervention effect (with a large effect size) was found for soft-drink consumption: intervention children decreased their consumption of soft drinks over time, while control children increased their consumption. Furthermore, differences in time spent playing outside (short-term) almost reached significance (P = 0.063). At 12 months, statistically significant intervention effects were found on parent report of child’s sedentary behavior and playing outside: intervention children decreased their time spent on TV viewing and computer games more than control children, while intervention children spent more time playing outside than control children. The use of active transport almost reached significance (P = 0.058).

**Table 4:** Short- and long-term intervention effects on physical activity, sedentary activity and nutrition.

	T0 (N = 75^1^)		Change T0—T1 (N = 56^1^)				Change T0—T2 (N = 591)			
	Intervention	Control	Intervention	Control			Intervention	Control		
Variable	Mean±SD	Mean±SD	Mean±SD	Mean±SD	B (95% CI)	Cohen’s d	Mean±SD	Mean±SD	B (95% CI)	Cohen’s d
*Questionnaire data^2^*										
Sedentary behavior	13.81±6.78	13.52±6.82	-3.95±4.40	-2.35±4.15	-1.74 (-4.18, 0.70)	-0.31	-2.71±4.45	-0.49±4.98	-2.39* (-4.64, -0.14)	-0.33
Playing outside	5.07±3.88	7.26±4.71	3.70±4.89	1.18±6.16	2.85 (-0.16, 5.86)	0.56	1.19±3.67	-1.25±3.11	1.94* (0.04, 3.84)	0.55
Sports club	2.35±2.27	1.70±2.35	0.06±1.72	0.75±1.86	-0.79 (-1.79, 0.22)	-0.39	0.60±2.57	0.62±1.43	-0.71 (-1.83, 0.40)	-0.01
Active transport	1.82±1.43	2.17±1.47	0.18±1.22	-0.15±1.14	0.24 (-0.43, 0.92)	0.25	0.23±1.57	-0.04±0.82	0.97 (-0.03, 1.98)	0.21
*Accelerometer^3^*										
MVPA	49.88±16.65	51.23±18.37	2.69±13.61	2.94±21.12	1.92 (-7.32, 11.15)	-0.01	0.14±11.58	-4.17±18.47	5.15 (-2.37, 12.67)	0.26
Sedentary	405.68±63.74	425.64±71.37	1.85±45.21	5.76±70.24	-17.13 (-52.41, 18.16)	-0.06	26.42±57.72	46.87±66.34	-27.85 (-62.39, 6.68)	-0.3
*Nutrition*										
Breakfast^4^	6.70±1.02	6.86±0.48	0.13±1.17	0.04±0.60	-0.01 (-0.36, 0.33)	0.1	0.10±0.75	0.14±0.52	-0.02 (-0.14, 0.10)	-0.06
Snacks^4^	6.42±3.35	5.06±3.28	-1.58±2.00	-0.19±2.43	-0.54 (-1.61, 0.53)	-0.42	-1.38±2.46	0.35±2.67	-0.86 (-2.00, 0.27)	-0.53
Fruits^5^	7.18±3.56	8.11±3.98	0.97±3.12	-0.12±3.94	0.22 (-1.59, 2.03)	0.28	1.13±4.16	1.26±3.81	-1.02 (0.54, 1.18)	-0.04
Vegetables^6^	405.04±225.60	340.51±192.05	-13.22±112.50	-64.24±105.60	31.02 (-48.54, 110.58)	0.24	-17.31±153.15	-55.69±158.36	12.20 (-81.30, 105.70)	0.17
Soft drinks^7^	10.68±7.87	10.00±6.75	-2.48±5.66	1.73±7.00	-4.89** (-8.20, -1.58)	-0.67	-1.07±8.20	0.14±6.66	-1.33 (-4.97, 2.32)	-0.17
Water^7^	13.50±9.26	11.19±6.71	4.82±9.13	3.35±7.35	1.67 (-3.08, 6.42)	0.18	5.31±10.74	1.84±7.40	3.87 (-1.76, 9.50)	0.44

Note: T0 = baseline, T1 = 4 months after baseline, T2 = 12 months after baseline, SD = standard deviation, B = regression coefficient, CI = confidence interval, MVPA = Moderate to Vigorous Physical Activity.

^1^maximum number of respondents, numbers differ per outcome measure.

^2^outcomes measured in hours per week.

^3^outcomes measured in minutes per day.

^4^frequency per week.

^5^pieces per week.

^6^grams per week.

^7^glasses per week.

*P<0.05

**P<0.001.

#### Parenting behaviors

All statistically significant differences regarding parenting outcomes were in favor of the intervention condition (Table 5). Parents in the intervention condition slightly increased their responsibility regarding physical activity at 4 months follow-up, whereas parents in the control condition reduced this. At 12 months, intervention effects were visible on monitoring of food intake (medium effect size) and responsibility regarding nutrition (small effect size): intervention parents increased their monitoring of the child’s food intake compared to baseline and felt equally responsible compared to baseline, while control parents reduced their monitoring of the child’s food intake and felt less responsible for the child’s nutrition.

**Table 5:** Short- and long-term intervention effects on parenting measures: parenting behaviors

	T0 (N = 73^1^)		Change T0—T1 (N = 57^1^)			Change T0—T2 (N = 57^1^)				
	Intervention	Control	Intervention	Control			Intervention	Control		
Variable^2^	Mean±SD	Mean±SD	Mean±SD	Mean±SD	B (95% CI)	Cohen’s d	Mean±SD	Mean±SD	B (95% CI)	Cohen’s d
*Parenting practices*										
Monitoring food intake	4.31±0.58	4.34±0.54	0.09±0.51	-0.06±0.45	0.14 (-0.11, 0.39)	+0.30	0.11±0.60	-0.15±0.41	0.26* (0.03, 0.49)	+0.46
Perceived responsibility regarding nutrition	4.32±0.53	4.28±0.62	-0.17±0.48	0.04±0.66	-0.17 (-0.47, 0.13)	-0.39	0.01±0.65	-0.20±0.65	0.33* (0.02, 0.64)	+0.32
Monitoring physical activity	4.31±0.58	4.34±0.54	0.09±0.51	-0.06±0.45	0.08 (-0.21, 0.38)	+0.30	0.11±0.60	-0.15±0.41	0.31 (-0.08, 0.71)	+0.50
Perceived responsibility regarding physical activity	3.76±0.97	3.93±0.84	0.05±0.77	-0.37±0.56	0.40*(0.08, 0.73)	+0.45	0.17±0.93	-0.10±0.71	0.30 (-0.13, 0.73)	+0.24
*Feeding styles*										
Control over eating	4.20±0.41	4.15±0.41	0.08 ±0.25	-0.07±0.32	0.09 (-0.07, 0.25)	+0.37	0.00±0.36	0.00±0.33	-0.02 (-0.21, 0.17)	+0.00
Instrumental feeding	1.76±0.67	1.89±0.65	-0.15±0.53	-0.23±0.47	0.08(-0.18, 0.33)	+0.13	-0.17±0.48	-0.16±0.48	-0.07 (-0.32, 0.18)	-0.02
Emotional feeding	1.41±0.54	1.49±0.52	-0.07±0.38	-0.13±0.42	0.07(-0.14, 0.28)	+0.11	-0.04±0.31	-0.19±0.46	0.07(-0.12, 0.26)	+0.28
Encouragement to eat	3.64±0.61	3.68±0.71	0.20±0.38	-0.02±0.35	0.24** (0.06, 0.41)	+0.37	0.12±0.45	0.17±0.45	0.05(-0.17, 0.28)	-0.08
*Parenting styles*										
Authoritative parenting	4.10±0.42	4.16±0.32	0.11±0.25	-0.02±0.25	0.08(-0.05, 0.20)	+0.40	0.28±0.34	0.29±0.37	-0.01(-0.17, 0.16)	-0.03
Authoritarian parenting	2.96±0.51	3.06±0.54	-0.07±0.34	-0.07±0.37	-0.01(-0.21, 0.18)	+0.00	-0.06±0.35	-0.14±0.33	0.06 (-0.14, 0.26)	+0.15
Psychological control	1.73±0.45	1.70±0.47	-0.16±0.30	0.07±0.37	-0.18*(-0.34, -0.01)	-0.50	-0.10±0.46	0.06±0.44	-0.17 (-0.42, 0.07)	-0.35
Efficacy of and satisfaction with parenting	4.79±0.53	4.82±0.48	0.13±0.37	-0.14±0.42	0.28*(0.06, 0.49)	+0.57	0.22±0.44	0.10±0.36	0.16(-0.05, 0.36)	+0.24

Note: T0 = baseline, T1 = 4 months after baseline, T2 = 12 months after baseline, SD = standard deviation, B = regression coefficient, CI = confidence interval.

^1^Maximum number of respondents, numbers differ per outcome measure.

^2^Scores on 5-point Likert scale 0–5.

*P<0.05

**P<0.001.

Change in the amount of encouragement to eat differed significantly between the groups at 4 months (medium effect size): intervention parents increased their encouragement of food variety and interest in food, while control parents did not change. This difference in change was no longer visible at 12 months after baseline. Differences in control over eating, instrumental feeding, and emotional feeding were not statistically significant.

At 4 months, intervention effects regarding parenting styles were visible on psychological control (medium effect size) and efficacy of and satisfaction with parenting (large effect size): intervention parents reduced their psychological control and increased their perceived efficacy and satisfaction regarding parenting compared to baseline, while the opposite was seen for control parents. These intervention effects were no longer statistically significant at 12 months. No significant differences in change were found with regard to authoritative and authoritarian parenting.

### Process evaluation

The majority of the group sessions which were planned actually took place: only 2 of the 60 group sessions were cancelled due to holidays or due to absence of the majority of the parents. Thirty percent (N = 13; nonselective regarding demographics) of the parents of children who were assigned to the intervention condition did not attend any intervention session at all. Of the parents who did visit at least one group session, 81% (parents of 25 children) were present at 5 or more sessions. In 80% of these children, one parent attended the group sessions and for 20% of these children both parents attended most sessions. Furthermore, parents who were attending the group sessions received at least 2 of 4 telephone sessions.

Parents who participated in the intervention were satisfied, indicated by the mean score of 66.67 (SD = 10.57) on the Client Satisfaction Questionnaire. In general, parents had a good impression of the program (Mean = 4.04, SD = 0.66), perceived the program as quite interesting (Mean = 4.12, SD = 0.77) and perceived the program as quite instructive (Mean = 4.15, SD = 0.73). Parents rated the value of the intervention as 7.7 (SD = 1.03) on a 10-point scale (84% ≥7).

## Discussion

The current study evaluated the effectiveness of a general parenting intervention, Lifestyle Triple P, aimed at preventing childhood obesity. Lifestyle Triple P is a derivative of the Triple P Positive Parenting Program [42], a multi-level parenting and family support strategy. Results of the current study showed no beneficial effect of this intervention on children’s BMI z-score, waist circumference, and skin-folds, compared to the control condition, neither immediately after the intervention (4 months) nor in the longer term (12 months). We did find short-term intervention effects on parent’s report of children’s soft-drink consumption, and parental responsibility regarding physical activity, encouragement to eat, psychological control, and efficacy and satisfaction with parenting. Longer-term intervention effects were found on parent’s report of children’s time spent on sedentary behavior (TV viewing and computer games), time spent on playing outside, and parental monitoring of food intake, and responsibility regarding nutrition. No significant effects were found for children’s objectively measured physical activity.

Several explanations can be given for the apparent lack of effects on the primary outcome measure. The Lifestyle Triple P intervention is a behavioral intervention which aims to change behavioral *determinants* (parenting behavior), in order to influence *behavior* (children’s nutrition and physical activity level), thereby finally affecting children’s *weight development*. Thus, the causal chain in our effectiveness study is rather long. This could explain why we did find some changes in behavior as a result of the intervention, whereas these changes did not, or at least not yet, result in improvements in children’s weight status and body composition. It is conceivable that if these behavioral changes are sustained over a longer time (>12 months), changes in children’s weight development may still occur. Note that some of the intervention effects we found at 4 months were not visible anymore at 12 months.

Some intervention components could however be further optimized. The recipes provided were found to be quite difficult for parents and not always appropriate to the Dutch eating habits. Furthermore, parents indicated that they would have liked a booster session (for example after 6 months) to refresh their knowledge and skills. Although Golan and colleagues [43] found better outcomes when parents were treated alone and children were not included in intervention sessions, it is worth considering whether children should be involved in the intervention.

Note that a small percentage of the children (17%) had a normal weight at baseline. These children were overweight or obese at the moment they were included, but turned to the upper range of a normal weight status score. We decided not to exclude these children from the study as prevention of excessive weight gain is also highly relevant for these children (i.e. not returning to the overweight status), and every child may benefit form an intervention aimed at improving parenting, diet and physical activity levels.

Results of intervention approaches similar to Lifestyle Triple P to date have been mixed; some studies found promising effects [12–14,17,44,45], while others found no effect on primary outcomes [14,15,46]. The efficacy of the Lifestyle Triple P intervention was tested previously in an RCT with a wait-list control condition, by West and colleagues [17]. These authors reported positive intervention effects on weight-related outcomes. However, comparison between this and our study is hampered by differences in the control conditions (wait-list control vs. control intervention). Trials using wait-list control conditions are more likely to show between group differences, but trials using a control intervention may provide an underestimation of the total effect of the intervention [47]. Also, the study sample used by West et al. was, on average, more obese than our sample (mean BMI z-scores 2.11 vs. mean BMI z-scores 1.85), which probably gives more room for improvement on BMI measures. Furthermore, the children in the study of West et al. were generally older (8.5 years vs. 7 years) which limits study comparability, due to differences in parental influence and differences in growth patterns at various ages. Finally, the Australian study was implemented as an efficacy study, while in the Dutch trial we tried to implement in the real life situation, which may have led to less significant study results [48].

### Strengths and limitations

Strengths of the current study include the RCT design with concealed group allocation, long-term follow-up (12 months after baseline), and high retention rates, which were similar for both conditions (80% at 12 months). Furthermore, the quality of the objective measurements was high: all anthropometric measurements were standardized and performed by the same trained research assistant who was blinded to the research condition, preventing measurement bias. Although not all questionnaires were validated in the Dutch language specifically, we mainly used validated questionnaires, and we used accelerometry for objective physical activity assessment. The intervention was based both on theory and evidence, and was highly appreciated by the parents who participated in the group sessions. Quality of the delivery of the intervention was assured by training and accreditation of facilitators, ongoing supervision, and manuals.

Limitations include the relatively small sample size, although we achieved enough power according to our sample size calculation (see Gerards et al. [18]). We put a lot of effort into the recruitment of parents [49] and recruitment took longer than planned. Recruiting parents has been shown to be a challenge in other intervention studies as well, thereby posing a threat to the generalizability of the study findings. We exclusively used validated measurements to assess parent reports of child behaviors and parenting. The ‘Physical Activity-Related Parenting Questionnaire’ has however not been validated and we used only a subset of items from a validated Food Frequency Questionnaire. This food frequency questionnaire had shown to be reliable in prior research [26]. We have included only two scales (monitoring and perceived responsibility) of the CFQ, because the empirical data regarding the desirability of pressure and restriction is still inconclusive (e.g. [50]). Moreover, although we randomly divided the participants into intervention or control condition, we are aware of some baseline differences between both groups, for example BMI of mothers. However, all analyses were adjusted for relevant confounders. Another limitation concerns the presence of missing values, which limited the possibility to conduct intention-to-treat analyses [51]. In order to overcome this problem, we applied a multiple imputation approach [41] to treat missing values. Since the use of multi-level analysis of multiple imputation data is still under-investigated, more research is necessary in this area in order to provide reliable estimates [41]. Consequently, we performed multiple linear regression analyses on the data in which the multiple imputation approach was used to treat missing values. This approach yielded effects in the same direction as the complete-case analyses, but the effects were non-significant under listwise deletion, see S1, S2, and S3 Tables.

## Conclusion

Although the Lifestyle Triple P intervention showed positive effects on some parent reported child behaviors and parenting measures, no effects were found on children’s body composition or objectively measured physical activity at 4 and 12 months after baseline.

## Supporting Information

S1 Checklist CONSORT 2010 checklist of information to include when reporting a randomised trial. Checklist of RCTs and where the topics are reported in the manuscript.
(DOC)

S1 Protocol Effectiveness of Lifestyle Triple P: an intervention aimed at the prevention of excessive weight gain in 4 to 8-year-old overweight children. Research protocol of the effectiveness study.
(DOC)

S1 Table Short- and long-term intervention effects on anthropometric outcomes (after multiple imputation). Analyses using a multiple imputation approach for treating missing data, effects on anthropometric outcomes.
(DOCX)

S2 Table Short- long term intervention effects on physical activity, sedentary activity and nutrition (after multiple imputation). Analyses using a multiple imputation approach for treating missing data, effects on nutrition and activity.
(DOCX)

S3 Table Short- and long-term intervention effects on parenting measures: parenting behaviors (after multiple imputation). Analyses using a multiple imputation approach for treating missing data, effects on parenting behaviors.
(DOCX)
