# Supplementary material for: The Effectiveness of Lifestyle Triple P in the Netherlands: A Randomized Controlled Trial
Source: PLoS One. 2015 Apr 7;10(4):e0122240. doi: 10.1371/journal.pone.0122240 (PMC4388496; doi:10.1371/journal.pone.0122240)
Supplement: S1 Protocol — Research protocol of the effectiveness study. (DOC) [file pone.0122240.s002.doc]

**Effectiveness of Lifestyle Triple P:**

**an intervention aimed at the prevention of excessive weight gain in 4 to 8-year-old overweight children**

***Onderzoek naar een programma om overmatige gewichtstoename bij 4 t/m 8-jarige kinderen tegen te gaan***

**(October 2011)**

**Effectiveness of Lifestyle Triple P: an intervention aimed at the prevention of excessive weight gain in 4 to 8-year-old overweight children**

| Protocol ID | MEC 10-3-052 |
| --- | --- |
| Short title | Effectiveness Lifestyle Triple P |
| Version | 1.3 October 2011 |
| Date | October 2011 |
| Coordinating investigator/project leader | Name: Dr. S.P.J. Kremers  Position: Associate Professor  Department: Health Promotion  Institute: Maastricht University  Address: P. Debyeplein1, 6229 HA, Maastricht  Telephone: 043-3882431  E-mail: s.kremers@maastrichtuniversity.nl |
| Principal investigator(s) (in Dutch: hoofdonderzoeker/uitvoerder)  *Multicenter research: per site* | Name: Dr. S.P.J. Kremers  Position: Associate Professor  Department: Health Promotion  Institute: Maastricht University  Address: P. Debyeplein1, 6229 HA, Maastricht  Telephone: 043-3882431  E-mail: s.kremers@maastrichtuniversity.nl |
| Sponsor (in Dutch: verrichter/opdrachtgever) | Maastricht University  Address: P.O. Box 616, 6200 MD, Maastricht |

| Independent physician(s) | Dr. J.W.M. Muris  Huisartsgeneeskunde, Universiteit Maastricht  Tel: 043-3882310  Email: [jean.muris@hag.unimaas.nl](mailto:jean.muris@hag.unimaas.nl) |
| --- | --- |
|  |  |
|  |  |

PROTOCOL SIGNATURE SHEET

| Name | Signature | Date |
| --- | --- | --- |
| Head of Department:  Prof. dr. N.K. De Vries  Head of Department of Health Promotion |  |  |
| Coordinating Investigator/Project leader/Principal Investigator:  Dr. S.P.J. Kremers  Associate Professor |  |  |

**TABLE OF CONTENTS**

INTRODUCTION AND RATIONALE [11](#__RefHeading___Toc262738571)

OBJECTIVES [13](#__RefHeading___Toc262738573)

1. STUDY DESIGN [13](#__RefHeading___Toc262738574)

STUDY POPULATION [13](#__RefHeading___Toc262738575)

1.1 Population (base) [13](#__RefHeading___Toc262738576)

1.2 Inclusion criteria [13](#__RefHeading___Toc262738577)

1.3 Exclusion criteria [13](#__RefHeading___Toc262738578)

1.4 Sample size calculation [13](#__RefHeading___Toc262738579)

2. TREATMENT OF SUBJECTS [13](#__RefHeading___Toc262738580)

2.1 Intervention [13](#__RefHeading___Toc262738581)

2.2 Use of co-intervention (if applicable) [13](#__RefHeading___Toc262738582)

2.3 Escape medication (if applicable) [13](#__RefHeading___Toc262738583)

3. METHODS [13](#__RefHeading___Toc262738584)

3.1 Study parameters/endpoints [13](#__RefHeading___Toc262738585)

3.1.1 Main study parameter/endpoint [13](#__RefHeading___Toc262738586)

3.1.2 Secondary study parameters/endpoints (if applicable) [13](#__RefHeading___Toc262738587)

3.1.3 Other study parameters (if applicable) [13](#__RefHeading___Toc262738588)

3.2 Randomisation, blinding and treatment allocation [13](#__RefHeading___Toc262738589)

3.3 Study procedures [13](#__RefHeading___Toc262738590)

3.4 Withdrawal of individual subjects [13](#__RefHeading___Toc262738591)

3.4.1 Specific criteria for withdrawal (if applicable) [13](#__RefHeading___Toc262738592)

3.5 Replacement of individual subjects after withdrawal [13](#__RefHeading___Toc262738593)

3.6 Follow-up of subjects withdrawn from treatment [13](#__RefHeading___Toc262738594)

3.7 Premature termination of the study [13](#__RefHeading___Toc262738595)

4. SAFETY REPORTING [13](#__RefHeading___Toc262738596)

4.1 Section 10 WMO event [13](#__RefHeading___Toc262738597)

4.2 Adverse and serious adverse events [13](#__RefHeading___Toc262738598)

4.2.1 Suspected unexpected serious adverse reactions (SUSAR) [13](#__RefHeading___Toc262738599)

4.2.2 Annual safety report [13](#__RefHeading___Toc262738600)

4.3 Follow-up of adverse events [13](#__RefHeading___Toc262738601)

4.4 Data Safety Monitoring Board (DSMB) [13](#__RefHeading___Toc262738602)

N.a.STATISTICAL ANALYSIS [13](#__RefHeading___Toc262738603)

STATISTICAL ANALYSIS [13](#__RefHeading___Toc262738604)

5. ETHICAL CONSIDERATIONS [13](#__RefHeading___Toc262738605)

5.1 Regulation statement [13](#__RefHeading___Toc262738606)

5.2 Recruitment and consent [13](#__RefHeading___Toc262738607)

5.3 Objection by minors or incapacitated subjects (if applicable) [13](#__RefHeading___Toc262738608)

5.4 Benefits and risks assessment, group relatedness [13](#__RefHeading___Toc262738609)

5.5 Compensation for injury [13](#__RefHeading___Toc262738610)

5.6 Incentives (if applicable) [13](#__RefHeading___Toc262738611)

6. ADMINISTRATIVE ASPECTS AND PUBLICATION [13](#__RefHeading___Toc262738612)

6.1 Handling and storage of data and documents [13](#__RefHeading___Toc262738613)

6.2 Amendments [13](#__RefHeading___Toc262738614)

6.3 Annual progress report [13](#__RefHeading___Toc262738615)

6.4 End of study report [13](#__RefHeading___Toc262738616)

6.5 Public disclosure and publication policy [13](#__RefHeading___Toc262738617)

7. REFERENCES [13](#__RefHeading___Toc262738618)

**LIST OF ABBREVIATIONS AND RELEVANT DEFINITIONS**

| ABR | ABR form, General Assessment and Registration form, is the application form that is required for submission to the accredited Ethics Committee (In Dutch, ABR = Algemene Beoordeling en Registratie) |
| --- | --- |
| AE | Adverse Event |
| AR | Adverse Reaction |
| BMI | Body Mass Index |
| CA | Competent Authority |
| CCMO | Central Committee on Research Involving Human Subjects; in Dutch: Centrale Commissie Mensgebonden Onderzoek |
| CV | Curriculum Vitae |
| DSMB | Data Safety Monitoring Board |
| EU | European Union |
| EudraCT | European drug regulatory affairs Clinical Trials |
| GCP | Good Clinical Practice |
| IB | Investigator’s Brochure |
| IC | Informed Consent |
| IMP | Investigational Medicinal Product |
| IMPD | Investigational Medicinal Product Dossier |
| METC | Medical research ethics committee (MREC); in Dutch: medisch ethische toetsing commissie (METC) |
| RCT | Randomized Controlled Trial |
| (S)AE | (Serious) Adverse Event |
| SPC | Summary of Product Characteristics (in Dutch: officiële productinfomatie IB1-tekst) |
| Sponsor | The sponsor is the party that commissions the organisation or performance of the research, for example a pharmaceutical  company, academic hospital, scientific organisation or investigator. A party that provides funding for a study but does not commission it is not regarded as the sponsor, but referred to as a subsidising party. |
| SUSAR | Suspected Unexpected Serious Adverse Reaction |
| Triple P | Positive Parenting Program |
| Wbp | Personal Data Protection Act (in Dutch: Wet Bescherming Persoonsgevens) |
| YHC | Youth Health Care |
|  |  |
| WMO | Medical Research Involving Human Subjects Act (in Dutch: Wet Medisch-wetenschappelijk Onderzoek met Mensen |
| ZonMW | The Netherlands Organisation for Health Research and Development |

**SUMMARY**

**Rationale:** Overweight and obesity are already prevalent in 4 to 8-year-old children. Obesity-inducing behaviours in young children have been found to track throughout the life span. Acknowledging the lack of effective interventions aimed at children aged 4 to 8-year-old, the current study aims at executing an effectiveness study in which a lifestyle intervention aimed at the parents of 4 to 8-year-old children is tested.

**Objective**: The main objective is to evaluate the effectiveness of Lifestyle Triple P.

**Study design:** The design of the current study is a Randomized Controlled Trial (RCT)

**Study population:** The research population consists of 288 participants (96 overweight and obese 4 to 8-year-old children and their parents).

**Intervention (if applicable)**: The lifestyle intervention, called Lifestyle Positive Parenting Program (Lifestyle Triple P), is aimed at parents of overweight and obese children. The intervention will take 14 weeks, consisting of eight weekly 120-minute parental group training sessions, two individual telephone sessions, one parental group training sessions, two individual telephone sessions and a final parental group training session. Triple P is an evidence-based preventively oriented parenting and family support strategy, based on social learning principles. Active skills training methods are employed to help parents acquire new knowledge and skills. The control condition receives two information leaflets (one about healthy nutrition and physical activity, and one about positive parenting), a Time Machine (an electronic screen time manager) and a web-based tailored feedback about providing a good parental example for your child regarding diet and physical activity.

**Main study parameters/endpoints:** The main study parameter is children’s body composition, determined by BMI z-score (calculated by weight, height, age and gender), waist circumference, and fat mass (assessed by biceps and triceps skinfold and deuterium dilution). Secondary study parameters are children’s lifestyle (snacking behaviour, soft-drink consumption, fruit-and vegetable consumption and physical activity), parental body composition (BMI, parental fat mass), parental physical activity level, parenting self-efficacy and parenting style.

**Nature and extent of the burden and risks associated with participation, benefit and group relatedness:** The intervention which the parents of the children receive will take 14 weeks and consists of ten parental group training sessions, and four individual telephone sessions. Furthermore, the body composition of the children and parents (i.e. length, weight, waist circumference, biceps and triceps skinfolds) is measured, children and their parents are asked to wear an Actigraph accelerometer for one week and parents are asked to fill out a questionnaire at baseline, 14 weeks follow-up and 12 months follow-up. Additionally, children’s fat mass is also estimated at baseline and 12 months follow-up using deuterium, the gold standard. Participation in the current study is voluntary and without any risks.

# INTRODUCTION AND RATIONALE

Overweight and obesity are having an increasing global public health impact worldwide, also in the Netherlands (1). Overweight and obesity are already present in 4 to 8-year-old children (12.4% and 3.1% of the Dutch children respectively) (2), and young children already show obesity-inducing behaviours that may track throughout the lifespan (3, 4). Young children have a relatively short history of unhealthy habits, which makes it easier to change these behaviours than in an adult population. However, relatively few studies have been aimed at the implementation and effectiveness of obesity prevention interventions in children aged 4-8 years (5-8). An example of an obesity intervention which starts early in life is described in Abée et al. (9), in which parents of babies in the second week after birth receive recommendations related to stimulating motor development and increasing physical activity, from a nurse in the Well Baby Clinic. To date, the results of this intervention have not been published (9).

# A home-based intervention targeting overweight children aged 4 to 8-years-old may help parents to learn how to provide a positive environment for their children (10, 11). Therefore, last year we implemented a home-based intervention based on the Positive Parenting Programme (Triple P) (12), which we tested in a pilot study (MEC 08-3-092). The Positive Parenting Program (Triple P) is a preventively-oriented parenting and family support strategy (12), which is based on social learning principles and is aimed at designing more positive environments for children. Triple P adopts a system-contextual or ecological perspective in supporting parents. The program aims to prevent severe behavioural, emotional and developmental problems in children by enhancing the knowledge, skills and confidence of parents. Triple P incorporates five levels of intervention on a tiered continuum of increasing strength for parents of children and adolescents. Level 1 mainly uses mass media resources to increase community awareness of parenting resources and the receptivity of parents to participating in programs. The intensity of the programme increases towards Level 5, which represents an enhanced behavioural family intervention program for families where child behaviour problems persist (12). Level 4 Triple P matches the aims of the current intervention, and was therefore applied. It consists of a parent training program for children with behavioural difficulties or who are at risk of developing such problems, which can be offered either individually or in a group of parents. The intervention we developed consisted thus of Level 4 Triple P with additional sessions focusing on environmental influences on energy balance-related behaviours in children.

In the pilot study (MEC 08-3-092) it was tested 1) whether this intervention was applicable to parents of 4-year-old overweight children and 2) whether the recruitment for the intervention was feasible for primary care youth health care (YHC) professionals to incorporate in their daily practice. The most important results were that:

1. Parents indicated in a focus group that they were very enthusiastic about the program, they liked the program sessions, felt comfortable in the group, they learned a lot and they reported that they already experienced a change in the behaviour of their child. However parents indicated that they preferred more sessions and more general information about healthy eating and physical activity.
2. YHC professionals faced various difficulties in the recruitment of overweight children via the Dutch YHC. As a result, we have adapted the recruitment procedure for the current study (see Study Design).

While we were conducting our pilot study, the results of an RCT performed by the University of Queensland evaluating the efficacy of Lifestyle Triple P, became available (13, 14). Lifestyle Triple P is very similar to the program we initially developed, it is also a modification of Level 4 Group Triple P, with additional sessions focusing on nutrition and physical activity, targeting families with overweight or obese children. Their study showed that Lifestyle Triple P resulted in increased parenting self-efficacy, reduced effective parenting and decreased children’s BMI and body fat of children aged 4 to 11-years old (13). We have studied the Lifestyle Triple P intervention materials in detail and we concluded that the intervention is very similar to the intervention we piloted. It differs from our pilot intervention on some points (i.e. more sessions, and more emphasis on knowledge transfer). These were exactly the points that were identified in our pilot study that could improve our intervention. It would be valuable to test whether the Lifestyle intervention is effective in the Netherlands (after translation).

The aim of the current study is therefore to test the effectiveness of Lifestyle Triple P in the Netherlands.

# OBJECTIVES

Primary Objective: The primary objective is to evaluate the effectiveness of the Lifestyle Triple P intervention in 4 to 8-year-old overweight children on their BMI z-score, and fat mass.

Secondary Objective(s): The secondary objectives of the current study are to determine the effect of the intervention on children’s lifestyle (snacking behaviour, soft-drink consumption, fruit-and vegetable consumption and physical activity), parental BMI, parental fat mass, parental physical activity level, parenting self-efficacy and parenting style.

# STUDY DESIGN

The design of the current study will be a Randomized Controlled Trial, in which the effectiveness of the Lifestyle Triple P intervention will be tested. The complete study will take 18 months.

In total, 288 parents and children will be recruited. Parents will be recruited in two ways: 1) via the Dutch Youth Health Care and 2) via mass media.

1) Parents of children who are, according to medical documents of the YHC (Municipal Health Service; GGD) overweight or obese (and there is no organic cause for the overweight) will be approached by telephone by health educators working in our project (also working as youth nurses at the YHC South-Limburg). In this telephone call, parents are provided with information about the intervention. In case parents prefer to get the information about the research face-to-face, a home visit can be planned. After the parents received oral information about the research, they also receive written information in the form of a patient information letter. Parents can decide their participation within two weeks. They confirm their participation by filling out an informed consent form.

This way of recruiting the parents fits within the standard procedures of the YHC. Following these standard procedures, the medical documents of children are screened for overweight and obesity using the weight-by height curve on the 1997 Dutch growth study charts. When the weight is ≥ 1 SDS, the BMI is calculated and by using the international BMI cut-off points of Cole et al. (15) (which are based on six large nationally representative cross sectional surveys on growth including the Netherlands), the child is labelled ‘normal weight’, ‘overweight, not obese’ or ‘obese’. Overweight and obese children are approached for an extra consult with the YHC physician. In our proposed recruitment strategy, they are contacted by the health educators of our project (youth nurses) to participate in the study. In case parents do not want to participate in the current study, they are still offered the extra consultation with the physician.

2) A website is developed where interested parents can apply for more detailed information and where they can register when they want to be approached for participation in the research. Through a mass media campaign, which consists of the dissemination of a brochure and a poster (via e.g., schools, day care centers, health centers), and advertisements in local newspapers, people will become aware of the program and the website. Families who register for participation in the program will by phoned by our health educators to provide them with more detailed information about the research. In case parents prefer to get the information about the research face-to-face, a home visit can be planned. After the telephone call or home visit, parents receive a patient information letter. Parents can decide their participation in the research within two weeks by filling out an informed consent form.

After they decided to participate in the research, participants (both parents and children) are asked to come to the Municipal Health Service (GGD) where baseline measurements are performed by the research team of the current study. Children and parents will be measured for their weight, height, skinfold thickness (biceps and triceps), and waist circumference. Materials and instructions for measuring deuterium dilution (used for measuring children’s body composition) will be provided to the parents, which they have to send back by mail within one week. Children and parents are asked to wear an Actigraph accelerometer for 7 days. Parents are asked to return a baseline questionnaire which their received one week earlier by mail.

After the baseline measurements, parents are randomly assigned to either the intervention or the control condition. Participants in the intervention condition receive a 14-week intervention which consists of ten parental group training sessions, and four individual telephone sessions for parents. Participants in the control condition receive two information leaflets (one about healthy nutrition and physical activity (Dutch Nutrition Centre; Voedingscentrum) and one about positive parenting (Netherlands Youth Institute; NJi)), a Time Machine (an electronic screen time manager), and web-based tailored feedback about providing a good parental example for your child regarding diet and physical activity (Dutch Nutrition Centre; Voedingscentrum).

Parents of both conditions receive a follow-up questionnaire by mail after the intervention. They are again asked to visit the Municipal Health Service (GGD) where the child and both parents will be measured for anthropometric measurements (not deuterium) by the research team of the current study. Parents and children will be asked to wear an Actigraph accelerometer for 7 days. A final visit where all measurements are conducted, will take place 12 months after baseline (see flow-chart). During this visit, again materials and instructions are provided for measuring deuterium dilution, which they have to send back by mail within one week.

Flow chart 1: RCT to test the effectiveness of Lifestyle Triple P aimed at the prevention of excessive weight gain in overweight children

| **Recruitment of participants** | |
| --- | --- |
| **Via youth health care** | **Via mass media** |
| Medical documents children (YHC) are screened to indicate overweight and obese children | Mass media makes people aware of the intervention and a website of the project |
| **↓** | **↓** |
|  | At the project website people are provided with information about the intervention. People can apply for more information about the intervention. |
|  | **↓** |
| Parents of children who are labeled as overweight, or obese are approached by telephone by a specialized health educator who provides information about the intervention to the parents | Parents of children who registered for more information are approached by telephone to assure that they are eligible for participation and to provide them with more information |
| **↓** | **↓**. |
| Optional: the health educator provides information during a home visit. | Optional: the health educator provides information during a home visit |
| **↓** | **↓** |
| Parents receive the patient information letter and informed consent form | Parents receive the patient information letter and informed consent form |
| **↓** | **↓** |
| If the parents are willing to participate (they have two weeks to decide), they are both asked to sign for informed consent | If the parents are still willing to participate(they have two weeks to decide), they are both asked to sign for informed consent |
| **↓** | **↓** |
| **Baseline measurements** | |
| **Parents** | **Children** |
| Baseline questionnaire parents, anthropometric measurements and Actigraph accelerometer | Anthropometric measurements (including deuterium dilution) and Actigraph accelerometer |
| **Randomization (on individual level) to one of the two conditions** | |
| **Intervention condition (N = 48)** | **Control condition (N = 48)** |
| 8 parental group training sessions (week 1-8)  **↓**  2 individual telephone sessions (week 9-10)  **↓**  1 parental group training sessions (week 11)  **↓**  2 individual telephone sessions (week 12-13)  **↓**  1 final parental group training session (week 14) | Parents receive a Time Machine, web-based tailored feedback about providing a good parental example for your child regarding diet and physical activity, and two leaflets: one about healthy nutrition and physical activity and one about positive parenting. |
| **Posttest measurements (immediately after the intervention, 14 weeks after baseline)** | |
| **Parents** | **Children** |
| Follow-up questionnaire, follow-up anthropometric measurements and Actigraph accelerometer | Follow-up anthropometric measurements (not deuterium dilution) and Actigraph accelerometer |
| **Posttest measurements (12 months after baseline)** | |
| **Parents** | **Children** |
| Follow-up questionnaire parents, follow-up anthropometric measurements and Actigraph accelerometer | Follow-up anthropometric measurements (including deuterium dilution) and Actigraph accelerometer |

# STUDY POPULATION

## Population (base)

The research population will consist of 96 child-parents triads (total 288 participants). Parents of children aged 4 to 8 years are eligible for participating when their child is considered ‘overweight or obese’. The sample is expected to include relatively many children of low socio-economic status and ethnic minorities. This sample is drawn from medical reports of 2600 children in the Westelijke Mijnstreek and Eastern South Limburg, who are aged 4-8 years. Of these children, about 12.4% is overweight and 3.1% is obese, and 50% is expected to participate in the program. Therefore 9 months are planned to recruit parents of 96 4 to 8-year-old children

## Inclusion criteria

Parents are eligible for inclusion when: 1) their child is aged 4 to 8-years-old, and 2) when their child is labelled as overweight or obese after calculating the BMI and by using the international cut-off points of Cole et al. (15) (which are based on six large nationally representative cross sectional surveys on growth including the Netherlands). Parents who agree to participate in the study, and both sign for informed consent, are included in the study.

## Exclusion criteria

Children who have a medical cause for their overweight and children of parents who both do not speak the Dutch language are excluded from the current study.

## Sample size calculation

The expected difference between the intervention and control condition is 0.30 BMI-points over 6 months (based on Robinson (16)). This difference would imply and approximated relative weight loss of 1480 grams per year in the experimental condition (1020 grams weight gain versus 2500 grams weight gain in the control condition). Maintained behavioural changes would then lead a child in the experimental condition to a weight corresponding to the 50^th^ percentile when it reaches the age of eight years.

The expected difference between both conditions would require a sample of 38 families in each condition to detect this difference with a power of .90 and p<.05. Adjusting for attrition, a total of 96 families will be randomly assigned to the intervention of control condition. Given an expected 10% of children to meet the inclusion criteria, 2600 4-year-olds that are aged 4-years old, and 50% participation-rate, recruitment is expected to take 9 months. Unless conflicting results from the pilot study, group sessions will consist of 8 families, resulting in 6 groups in the intervention condition.

# TREATMENT OF SUBJECTS

## Intervention

Forty-eight parent pairs who are assigned to the intervention condition will receive a 14-week intervention which consists of eight 120-minute parental group training sessions, two 15-30-minute individual telephone session for parents, one 120-minute parental group training session, another two 15-30-minute individual telephone session for parents and a final 120-minute group training session.

The health educators who provide training to the parents have followed an official 3-day Triple P level 4 training by the Dutch Youth Institute (Nederlandse Jeugd Instituut) and received accreditation for this training. In addition, before the start of the intervention, the health educators will be dedicated to childhood overweight-related topics.

The group size for the group sessions will be formed by the parents of eight children. The methodology will follow the principles of Lifestyle Triple P, an intervention which was originally developed and tested at the Queensland University in Australia. A yet unpublished RCT showed that Lifestyle Triple P resulted in increased parenting self-efficacy, reduced effective parenting and decreased children’s BMI and body fat (13). Lifestyle Triple P consists of the Triple P intervention, which is used worldwide to manage behavioural problems among children (17), with additional sessions focusing on environmental influences on energy balance-related behaviours in children. Active skills training methods are employed to help parents acquire new knowledge and skills: e.g. brief presentations alternated with group discussion, video and live demonstrations of parenting skills, rehearsal of skills using role plays and peer modeling, and small group problem solving exercises. Between sessions, parents complete homework tasks in order to apply new knowledge and skills in their own home situation. Each family is provided with a parent workbook that contains all the information and activities presented in the sessions. An overview of the topics and strategies is provided in figure 1.

Figure 1: Overview of the topics and strategies per session

| **Session** | **Topics** | **Strategies** |
| --- | --- | --- |
| Session 1: Preparing for change | Nature and causes of obesity  Overview of Lifestyle Triple P  Readiness to change |  |
| Session 2: Understanding nutrition | Increasing children’s self esteem  Food groups and daily severs  Nutrition goals | Spend quality time with your child  Talk to your child  Show affection |
| Session 3: Understanding physical activity | Encouraging healthy behaviours  Physical activity goals  Increasing incidental activity  Reducing sugar intake | Give frequent praise  Set a good example  Replace foods high in added sugar  Provide water as a regular drink  Make family leisure time active  Encourage active transport |
| Session 4: Using rewards and modifying recipes | Using behaviour charts  Reducing fat intake  Modifying recipes | Use behaviour charts  Buy low fat foods  Use low fat cooking methods  Replace high fat ingredients |
| Session 5: Limiting sedentary activity and reading food labels | Limiting sedentary activities  Establishing ground rules  Reading food labels | Establish clear ground rules  Use directed discussion to deal with rule-breaking  Read food labels  Limit sedentary activities |
| Session 6: Playing active games | Providing active alternatives  Increasing movement skills | Provide active games  Play with your child |
| Session 7: Providing healthy meals | Establishing eating routines  Providing healthy meals/snacks  Children’s participation in sport | Establish eating routines  Provide healthy meals and snacks  Encourage participation in sport |
| Session 8: Managing problem behaviour | Managing problems behaviours | Use planned ignoring for minor misbehaviour  Give clear calm instructions  Back up instructions with logical consequences, quiet time, or time-out |
| Session 9: Using Lifestyle Triple P strategies 1 | Implementing strategies |  |
| Session 10: Using Lifestyle Triple P strategies 2 | Implementing strategies |  |
| Session 11: Planning ahead | Family survival tips  High risk situations  Planned activities routine |  |
| Session 12: Using planned activities 1 | Implementing planned activities routine |  |
| Session 13: Using planned activities 2 | Implementing planned activities routine |  |
| Session 14: Program Close | Progress review  Maintaining changes  Problem solving for the future |  |

Forty-eight parents who are assigned to the control condition receive an intervention which consists of three components. They receive:

1) two information leaflets: one about healthy nutrition and physical activity (Netherlands Institute for Sports and Physical Activity; NISB), and one about positive parenting of the Netherlands Youth Institute (NJi) information leaflets),

2) web-based tailored feedback about providing a good parental example for your child regarding diet and physical activity (Dutch Nutrition Centre; Voedingscentrum)

3) an electronic screen time manager (Time Machine) (16). The Time Machine is an automated device that can control and monitor the use of televisions, and television-based sedentary activities.

## Use of co-intervention (if applicable)

N.a.

## Escape medication (if applicable)

N.a.

INVESTIGATIONAL MEDICINAL PRODUCT

N.a.

# METHODS

## Study parameters/endpoints

### Main study parameter/endpoint

The primary objective of this study is to test the effectiveness of the intervention in terms of preventing overweight and obese children from excessive weight gain. The main study parameter is children’s body composition, determined by BMI z-score (calculated by weight, height, age and gender), waist circumference, and fat mass (assessed via biceps and triceps skinfold and deuterium). BMI, waist circumference, and skinfold is measured at baseline, 14 weeks (directly following the intervention) and 12 months follow-up. Deuterium is measured only at baseline and 12 months follow-up.

### Secondary study parameters/endpoints (if applicable)

Secondary study parameters are parental BMI and fat mass (assessed via biceps and triceps skinfold), parenting skills, the parental self-efficacy, children’s lifestyle (screen-viewing behaviour, snacking behaviour, soft-drink consumption, F&V consumption and physical activity), and parental physical activity level. These data are assessed at baseline, 14 weeks follow-up and 12 months follow-up, via questionnaires, and the Actigraph accelerometers.

### Other study parameters (if applicable)

The following study parameters are taken into account as confounders: SES, ethnicity, and children’s temperament, which are all measured in a baseline questionnaire.

## Randomisation, blinding and treatment allocation

Block randomisation (n = 4) will be performed on the individual level. After parents of four families decide to participate in the study, they will be randomised to either the experimental condition or the control condition.

## Study procedures

The overweight children and their parents will undergo a set of measurements at baseline, immediately following the intervention at 14 weeks and at 12 months follow-up. The following measurements are performed (see flow chart):

- Anthropometric measurements:
  - Weight and height (child and parents) to calculate BMI and BMI z-score
  - Biceps and triceps skinfold thickness (child and parents) using the Harpenden skinfold calliper to indicate changes in fat mass
  - Waist circumference
  - Deuterium (only children) to indicate fat mass (at baseline and 6 months)
- Actigraph accelerometer (child and parents)
- Questionnaire (parents, see appendix) measuring the following constructs:
  - Demographic variables
  - Physical activity child and screen-viewing behaviour
  - Snacking behaviour, soft-drink consumption child, fruit-and vegetable consumption child
  - Children’s behaviour questionnaire measuring children’s temperament
  - Parenting practices:
    - - Child feeding behaviour questionnaire (Birch) and child physical activity behaviour
      - Parental feeding style questionnaire (Wardle)
  - Parenting self-efficacy 🡪 Being a Parent Scale
  - Parenting styles

The anthropometric measurements are conducted during a visit at the Municipal Health Service (GGD) in both parents and the child. At this visit, materials and instructions for executing deuterium dilution are distributed. Parents are instructed to collect a urine sample of their child at the evening before their child drinks deuterium enriched water. In the morning, after an overnight fast, the second morning urine should be collected as well. The urine samples are collected in small bottles, parents are asked to send them back in an envelope.

The intervention consists of ten 120-minute parental group training sessions and four individual telephone session for parents. The parental group training sessions and the individual telephone sessions will be guided by the same health educator.

## Withdrawal of individual subjects

Subjects can leave the study at any time for any reason if they wish to do so without any consequences. The investigator can decide to withdraw a subject from the study for urgent medical reasons.

### Specific criteria for withdrawal (if applicable)

N.a.

## Replacement of individual subjects after withdrawal

N.a.

## Follow-up of subjects withdrawn from treatment

N.a.

## Premature termination of the study

N.a.

# SAFETY REPORTING

## Section 10 WMO event

In accordance to section 10, subsection 1, of the WMO, the investigator will inform the subjects and the reviewing accredited METC if anything occurs, on the basis of which it appears that the disadvantages of participation may be significantly greater than was foreseen in the research proposal. The study will be suspended pending further review by the accredited METC, except insofar as suspension would jeopardise the subjects’ health. The investigator will take care that all subjects are kept informed.

## Adverse and serious adverse events

Adverse events are defined as any undesirable experience occurring to a subject during the study, whether or not considered related to [the investigational product / the experimental treatment]. All adverse events reported spontaneously by the subject or observed by the investiga­tor or his staff will be recorded.

A serious adverse event is any untoward medical occurrence or effect that at any dose:

- results in death;
- is life threatening (at the time of the event);
- requires hospitalisation or prolongation of existing inpatients’ hospitalisation;
- results in persistent or significant disability or incapacity;
- is a congenital anomaly or birth defect;
- is a new event of the trial likely to affect the safety of the subjects, such as an unexpected outcome of an adverse reaction, lack of efficacy of an IMP used for the treatment of a life threatening disease, major safety finding from a newly completed animal study, etc.

All SAEs will be reported through the web portal *ToetsingOnline* to the accredited METC that approved the protocol, within 15 days after the sponsor has first knowledge of the serious adverse reactions.

SAEs that result in death or are life threatening should be reported expedited. The expedited reporting will occur not later than 7 days after the responsible investigator has first knowledge of the adverse reaction. This is for a preliminary report with another 8 days for completion of the report.

### Suspected unexpected serious adverse reactions (SUSAR)

N.a.

### Annual safety report

N.a.

## Follow-up of adverse events

N.a.

## Data Safety Monitoring Board (DSMB)

# N.a.

# STATISTICAL ANALYSIS

Primary and secondary outcome parameters will be described via descriptive statistics. Quantitative continuous variables will be presented by use of means and standard deviations. Of categorical data, percentages will be presented of respondents falling in each of the possible categories.

Both univariate and multivariate multilevel analyses (correcting for group effects) are conducted to determine the effect of the intervention on (changes in) BMI z-score, fat mass, children’s lifestyle (screen-viewing behaviour, snacking behaviour, soft-drink consumption, fruit-and vegetable consumption, and physical activity level), parenting self-efficacy, parental skills, parental BMI, parental fat mass, and parental physical activity level. In the multilevel analyses, relevant confounders such as the child’s age, gender, ethnicity and parental socio-economic status.

# ETHICAL CONSIDERATIONS

## Regulation statement

The MEC of Maastricht University has to approve the study before it can be carried out. The study will be conducted according to the principles of the Declaration of Helsinki (October 2008) and in accordance with the Medical Research Involving Human Subjects Act (WMO).

## Recruitment and consent

Parents are orally informed about the research by health educators working in our project. In addition, parents receive a patient information letter. Parents have two weeks to decide whether they are willing to participate. They confirm their participation by filling out an informed consent form. In the informed consent form, participants will be asked whether they give consent to be approached for further research in the future. Participants know that they can leave the study whenever they want, without giving their reasons. Furthermore, in the consent form it is described that the privacy of the participants is protected, that their data will be coded before the analyses, and that the data will be stored for five years. Participants can indicate on the informed consent form whether they would like to be acquainted of the results of the study or not.

## Objection by minors or incapacitated subjects (if applicable)

When the children show objection for the anthropometric measurements or when they resist wearing the Actigraph accelerometer, the procedures will be stopped immediately (‘objection by minors’). Objection by minors is defined as the situation in which the behaviour of the child deviates or manifests more excessively to regular daily routine in deviating situations. The parents as well as the researcher are involved in the appraisal of the child’s behaviour. The appraisal of objection is a continuing process during the research.

## Benefits and risks assessment, group relatedness

The Lifestyle Triple P intervention is expected to lead to better parental skills, higher parental self-efficacy and a healthier lifestyle of the child. Furthermore, the intervention aims at decrease the children’s overweight status leading to health benefits and less health risks. The children themselves will not be included in the intervention. Participation in the study (for parents) is voluntarily and without any risk.

## Compensation for injury

The intervention entails no increased risk or injury for the children or for the parents, and therefore in our opinion it is not necessary to have a subject insurance (proefpersonenverzekering). The Medical Research Committee (MEC) azM / UM has agreed with such a procedure at 23-06-2010.

The liability insurance of Maastricht University is applicable to this study.

## Incentives (if applicable)

Participants will receive a compensation for their travelling costs.

# ADMINISTRATIVE ASPECTS AND PUBLICATION

## Handling and storage of data and documents

Signed informed consent forms will be stored separately in an archive. Only the project team will access to this archive. A subject identification code is used for all the collected data, so that the privacy of the subjects is supported. Only the project team knows the key to this code and has access to the source data.

Urine collected via deuterium dilution is stored in cold stores. Here the same identification codes are used.

## Amendments

Amendments are changes made to the research after a favourable opinion by the accredited METC has been given. All amendments will be notified to the METC that gave a favourable opinion.

## Annual progress report

The sponsor/investigator will submit a summary of the progress of the trial to the accredited METC once a year. Information will be provided on the date of inclusion of the first subject, numbers of subjects included and numbers of subjects that have completed the trial, serious adverse events/ serious adverse reactions, other problems, and amendments.

## End of study report

The investigator will notify the accredited METC of the end of the study within a period of 8 weeks. The end of the study is defined as the last home visit.

In case the study is ended prematurely, the investigator will notify the accredited METC, including the reasons for the premature termination.
 Within one year after the end of the study, the investigator/sponsor will submit a final study report with the results of the study, including any publications/abstracts of the study, to the accredited METC.

## Public disclosure and publication policy

The results of the study will be reported in national and international peer-reviewed scientific journals. The grant organisation will not impose any restrictions concerning publication of the study results. All results, also ‘negative’ results, will be published.

# REFERENCES

1. Visscher TLS, Seidell JC. The public health impact of obesity. Annual Review of Public Health. 2001;22:355-75.

2. Van den Hurk K, Van Dommelen P, De Wilde JA, Verkerk PH, Van Buuren S, Hirasing RA. Prevalentie van overgewicht en obesitas bij jeugdigen 4-15 jaar in de periode 2002-2004. Leiden: TNO Kwaliteit van Leven2006.

3. Margarey AM, Daniels LA, Boulton TJ, Cockinton RA. Predicting obesity in early adulthood from childhood and parental obesity. International Journal of Obesity. 2003;27:505-13.

4. Whitaker RC, Wright JA, Pepe MS, Seidel KD, Dietz WH. Predicting obesity in young adulthood from childhood and parental obesity. N Engl J Med. 1997 Sep 25;337(13):869-73.

5. Bluford DAA, Sherry B, Scanlon KS. Interventions to prevent or treat obesity in preschool children: a review of evaluated programs. Obesity. 2007;15(6):1356-72.

6. Campbell KJ, Hesketh KD. Strategies which aim to positively impact on weight, physical activity, diet and sedentary behaviours in children form zero to five years. A systematic review of the literature. Obesity Reviews. 2007;8:327-38.

7. Kremers S, Martens M, Reubsaet A, De Weerdt I, De Vries N, Jonkers R. Programmeringstudie Overgewicht.: ResCon en Universiteit Maastricht2008.

8. Doak CM, Visscher TL, Renders CM, Seidell JC. The prevention of overweight and obesity in children and adolescents: a review of interventions and programmes. Obes Rev. 2006 Feb;7(1):111-36.

9. L'Abee C, Sauer PJJ, Damen M, Rake JP, Cats H, Stolk RP. Cohort Profile: The GECKO Drenthe study, overweight programming during early childhood. International Journal of Epidemiology.

10. Harvey-Berino J, Rourke J. Obesity prevention in preschool native-american children: a pilot study using home visiting. Obesity Research. 2003;11(5):606-11.

11. Haire-Joshu D, Elliott MB, M. CN, Hessler K, Nanney MS, Hale N, et al. High 5 for Kids: The impact of a home visiting program on fruit and vegetable intake of parents and their preschool children. Preventive Medicine. 2008;47:77-82.

12. Sanders MR, Markie-Dadds C, Turner KMT. Theoretical, scientific and clinical foundations of the triple p-positive parenting program: a population approach to the promotion of parenting competence. Parenting Research and Practice Monograph. 2003;1:1-21.

13. West F. The Lifestyle Triple P Project. Exploring the link between parenting and childhood obesity: University of Queensland; 2007.

14. Service CaACH. Lifestyle Triple P Key Preliminary Findings: 2009 WA Community Health Pilot: Department of Health Western Australia2010.

15. Cole TJ, Bellizzi MC, Flegal KM, Dietz WH. Establishing a standard definition for child overweight and obesity worldwide: international survey. BMJ. 2000;320:1240-3.

16. Robinson TN. Reducing children's television viewing to prevent obesity. A randomized controlled trial. American Medical Association. 1999;282(16):1561-7.

17. De Graaf I, Speetjens P, Smit F, De Wolff M, Tavecchio L. Effectiveness of the triple p positive parenting program on behavioral problems in children; a meta-analysis. Behavior Modification. 2008;32:714-35.
