# Supplementary material for: The Effectiveness of Lifestyle Triple P in the Netherlands: A Randomized Controlled Trial
Source: PLoS One. 2015 Apr 7;10(4):e0122240. doi: 10.1371/journal.pone.0122240 (PMC4388496; doi:10.1371/journal.pone.0122240)
Supplement: S1 Table — Analyses using a multiple imputation approach for treating missing data, effects on anthropometric outcomes. (DOCX) [file pone.0122240.s003.docx]

**S1 Table** Short- and long-term intervention effects on anthropometric outcomes (after multiple imputation)

|  | T0 | | Change T0-T1 | | | | Change T0-T2 | |  |  |
| --- | --- | --- | --- | --- | --- | --- | --- | --- | --- | --- |
|  | Intervention | Control | Intervention | Control |  |  | Intervention | Control |  |  |
| Variable | Mean±SD | Mean±SD | Mean±SD | Mean±SD | B | Cohen’s *d* | Mean±SD | Mean±SD | B | Cohen’s *d* |
| BMI z-score | 1.82±0.83 | 1.86±0.74 | 0.03±0.25 | -0.01±0.21 | 0.012 | +0.05 | 0.29±0.44 | 0.05±0.34 | 0.127 | +0.30 |
| Waist circumference^1,2^ | 67.30±8.37 | 68.76±8.68 | 0.69±3.73 | 0.69±3.86 | -0.399 | 0.00 | 4.46±5.07 | 3.01±5.40 | 0.055 | +0.17 |
| Sum score skinfolds^1,3^ | 44.80±13.71 | 44.11±11.31 | -1.37±7.77 | -1.73±8.18 | -0.488 | +0.03 | 0.79±12.58 | -1.01±11.61 | 0.464 | +0.14 |

Note: T0=baseline, T1=4 months after baseline, T2=12 months after baseline, SD=standard deviation, B = regression coefficient; ^1^analyses corrected for child’s age and gender; ^2^waist circumference was measured in cm; ^3^skinfolds is the sum score of the biceps and triceps skinfolds, measured in mm; *P<0.05, **P<0.001
