# Supplementary material for: The Effectiveness of Lifestyle Triple P in the Netherlands: A Randomized Controlled Trial
Source: PLoS One. 2015 Apr 7;10(4):e0122240. doi: 10.1371/journal.pone.0122240 (PMC4388496; doi:10.1371/journal.pone.0122240)
Supplement: S2 Table — Analyses using a multiple imputation approach for treating missing data, effects on nutrition and activity. (DOCX) [file pone.0122240.s004.docx]

**S2 Table** Short- long term intervention effects on physical activity, sedentary activity and nutrition (after multiple imputation)

|  | T0 | | Change T0-T1 | |  |  | Change T0-T2 |  |  |  |  |
| --- | --- | --- | --- | --- | --- | --- | --- | --- | --- | --- | --- |
|  | Intervention | Control | Intervention | Control |  |  | Intervention | Control |  |  |  |
| Variable | Mean±SD | Mean±SD | Mean±SD | Mean±SD | B | Cohen’s *d* | Mean±sd | Mean±sd | B | Cohen’s *d* |  |
| *Questionnaire data*^1^ | | | | | | | | | | |  |
| Sedentary activity | 13.76±7.73 | 13.86±7.87 | -1.81±7.32 | -1.11±7.33 | -1.023 | -0.09 | -0.64±8.22 | 0.97±9.39 | -0.804 | -0.21 |  |
| Playing outside | 4.96±4.26 | 7.10±5.36 | 2.93±6.13 | 1.46±8.41 | 0.776 | +0.30 | 1.04±4.51 | -0.29±7.15 | 0.700 | +0.28 |  |
| Sport club | 2.86±3.28 | 1.96±2.65 | 0.14±3.23 | 1.04±3.10 | -0.632 | -0.30 | 0.74±4.95 | 1.61±4.59 | -0.360 | -0.29 |  |
| Active transport | 1.84±1.63 | 2.24±1.70 | -0.04±1.95 | -0.11±1.89 | 0.031 | +0.04 | 0.13±2.51 | -0.01±2.40 | 0.103 | +0.08 |  |
| *Actigraph accelerometer*^2^ | | | | | | | | | | |  |
| MVPA | 51.03±19.56 | 51.63±20.73 | 4.73±25.13 | 2.09±25.10 | 3.109 | +0.13 | 3.65±26.70 | -1.04±30.86 | 4.825 | +0.23 |  |
| Sedentary activity | 406.34±70.07 | 422.26±73.46 | 5.77±91.51 | 7.63±87.86 | -17.351 | -0.03 | 58.90±116.24 | 54.49±149.36 | -8.155 | +0.06 |  |
| *Nutrition* |  |  |  |  |  |  |  |  |  |  | |
| Breakfast^3^ | 6.46±1.34 | 6.65±0.97 | 0.12±1.42 | -0.09±1.19 | 0.049 | +0.18 | 0.39±1.29 | 0.14±0.98 | 0.079 | +0.21 | |
| Snacks^3^ | 6.62±3.66 | 5.40±4.04 | -1.46±2.60 | -0.76±3.20 | -0.122 | -0.18 | -1.65±3.73 | -0.24±4.12 | -0.719 | -0.37 | |
| Fruits^4^ | 7.30±4.82 | 8.27±5.22 | 0.59±4.46 | -0.10±5.11 | 0.203 | +0.14 | 1.11±6.27 | 1.45±7.47 | -1.524 | -0.07 | |
| Vegetables^5^ | 164.76±272.11 | 156.34±205.51 | -24.64±269.71 | -37.45±202.33 | 8.827 | +0.05 | 5.38±277.81 | -16.44±282.22 | 29.329 | +0.09 | |
| Softdrink^6^ | 10.52±8.36 | 10.47±9.09 | -1.69±9.05 | -0.14±11.31 | -2.564 | -0.18 | 0.64±11.25 | 1.58±14.12 | -0.772 | -0.11 | |
| Water6 | 13.72±13.13 | 11.78±9.83 | 3.19±14.30 | 2.81±14.66 | 1.002 | +0.03 | 3.68±17.32 | 1.84±13.98 | 3.109 | +0.16 | |

Note:T0=baseline, T1=4 months after baseline, T2=12 months after baseline, SD= standard deviation, B=regression coefficient, MVPA = Moderate- to Vigorous Physical Activity; ^1^outcomes measured in hours per week, ^2^outcomes measures in minutes per day, ^3^frequency per week, ^4^pieces per week, ^5^grams per week, ^6^glasses per week;*P<0.05, **P<0.001
