# Supplementary material for: The Effectiveness of Lifestyle Triple P in the Netherlands: A Randomized Controlled Trial
Source: PLoS One. 2015 Apr 7;10(4):e0122240. doi: 10.1371/journal.pone.0122240 (PMC4388496; doi:10.1371/journal.pone.0122240)
Supplement: S3 Table — Analyses using a multiple imputation approach for treating missing data, effects on parenting behaviors. (DOCX) [file pone.0122240.s005.docx]

**S3 Table** Short- and long-term intervention effects on parenting measures: parenting behaviors (after multiple imputation)

|  | T0 | | Change T0-T1 | |  |  | Change T0-T2 | |  |  |  |
| --- | --- | --- | --- | --- | --- | --- | --- | --- | --- | --- | --- |
|  | Intervention | Control | Intervention | Control | B | Cohen’s *d* | Intervention | Control | B | Cohen’s *d* |  |
| Variable^1^ | Mean±SD | Mean±SD | Mean±SD | Mean±SD |  |  | Mean±SD | Mean±SD |  |  |  |
| *Parenting practices* |  |  |  |  |  |  |  |  |  |  |  |
| Monitoring food intake | 4.26±0.68 | 4.29±0.64 | 0.08±0.81 | -0.05±0.80 | 0.113 | +0.20 | 0.08±0.79 | -0.12±0.72 | 0.211 | +0.30 |  |
| Responsibility regarding nutrition | 4.30±0.65 | 4.29±0.65 | -0.15±0.69 | -0.13±0.97 | -0.040 | -0.03 | -0.15±0.84 | -0.33±1.10 | 0.233 | +0.28 |  |
| Monitoring physical activity | 3.86±0.88 | 4.01±0.88 | 0.06±0.97 | -0.11±1.04 | 0.061 | +0.19 | 0.20±1.19 | 0.10±1.07 | 0.091 | +0.11 |  |
| Responsibility regarding physical activity | 3.76±0.99 | 3.90±1.03 | -0.04±1.23 | -0.30±1.16 | 0.215 | +0.26 | 0.04±1.47 | -0.15±1.53 | 0.204 | +0.19 |  |
| *Feeding style* |  |  |  |  |  |  |  |  |  |  |  |
| Control over eating | 4.19±0.45 | 4.14±0.48 | 0.05±0.39 | -0.13±0.50 | 0.181 | +0.39 | -0.05±0.60 | -0.16±0.74 | 0.113 | +0.24 |  |
| Instrumental feeding | 1.78±0.72 | 1.9± 0.68 | -0.12±0.56 | -0.23±0.66 | 0.053 | +0.16 | -0.15±0.62 | -0.11±0.79 | -0.091 | -0.06 |  |
| Emotional feeding | 1.44±0.63 | 1.52±0.60 | -0.04±0.53 | -0.03±0.58 | 0.008 | -0.02 | -0.08±0.59 | -0.09±0.77 | -0.010 | +0.02 |  |
| Encouragement to eat | 3.59±0.71 | 3.59±0.81 | 0.19±0.68 | 0.09±0.82 | 0.088 | +0.13 | 0.21±0.80 | 0.27±1.07 | 0.036 | -0.08 |  |
| *Parenting style* | |  |  |  |  |  |  |  |  |  |  |
| Authoritative parenting | | 4.10±0.44 | 4.13±0.42 | 0.07±0.33 | 0.02±0.51 | 0.027 | +0.12 | 0.30±0.49 | 0.37±0.55 | -0.011 | -0.16 |
| Authoritarian parenting | | 2.95±0.56 | 3.01±0.61 | -0.12±0.49 | -0.14±0.56 | 0.011 | +0.03 | -0.15±0.52 | -0.12±0.67 | 0.023 | -0.05 |
| Psychological control | | 1.69±0.49 | 1.71±0.53 | -0.10±0.43 | 0.06±0.71 | -0.143 | -0.31 | -0.12±0.59 | -0.02±0.77 | -0.165 | -0.20 |
| Efficacy and satisfaction parenting | | 4.81±0.59 | 4.78±0.55 | 0.02±0.69 | -0.14±0.57 | 0.211 | +0.28 | 0.12±0.60 | 0.08±0.72 | 0.121 | +0.07 |

Note: T0=baseline, T1=4 months after baseline, T2=12 months after baseline, SD=standard deviation, B=regression coefficient; ^1^scores on 5-point Likert scale 0-5; *P<0.05, **P<0.001
